# Description, identification, and growth of *Tuber borchii* Vittad. mycorrhized *Pinus sylvestris* L. seedlings on different lime contents

**DOI:** 10.1007/s00572-023-01135-3

**Published:** 2024-01-18

**Authors:** Tanja Mrak, Tine Grebenc, Silke Friedrich, Babette Münzenberger

**Affiliations:** 1 Department of Forest Physiology and Genetics, Slovenian Forestry Institute, Večna pot 2, 1000 Ljubljana, Slovenia; 2 Truffle Nursery, Schneckleinsberg 5, 91788 Pappenheim, Germany; 3 Department of Fungal Interactions, Research Area 1 ‘Landscape Functioning’, Leibniz Centre for Agricultural Landscape Research (ZALF), Eberswalder Strasse 84, 15374 Müncheberg, Germany

**Keywords:** Anatomy, Ectomycorrhiza, Limestone content, Molecular identification, *Pinus sylvestris*, *Tuber borchii*

## Abstract

*Tuber borchii* forms ectomycorrhiza with oaks, hazel, and pines, including *Pinus sylvestris*. However, its ectomycorrhiza morphotype with *P. sylvestris* was not comprehensively described so far, and molecular analyses are missing despite a high danger of misidentification of *T. borchii* ectomycorrhiza with other closely related and less valuable truffle species. We described for the first time the morphology and anatomy of *T. borchii-P. sylvestris* ectomycorrhiza using differential interference contrast technique and semi-thin sections in combination with molecular confirmation of identity. Color of ectomycorrhiza is reddish to dark brown, and morphotypes are unevenly but densely covered by warts-bearing pin-like cystidia. All layers of the hyphal mantle are pseudoparenchymatous with outer mantle layer formed of epidermoid cells. *T. borchii* ectomycorrhiza was identified by a molecular comparison with fruitbodies used for inoculation and its respective ectomycorrhizae. *T. borchii* has a wide ecological amplitude. To get a better insight in mycorrhization requirements, we investigated growth of *P. sylvestris* and its ectomycorrhiza infection rate with *T. borchii* in substrate with different lime content. The mycorrhization of *P. sylvestris* with *T. borchii* in the mycorrhization substrate and cultivation in greenhouse conditions was successful, with colonization of *P. sylvestris* varying between 36.5 and 48.1%. There was no significant correlation of mycorrhization to applied lime contents, and consequently to pH in substrate, while the increased levels of lime improved growth of the *P. sylvestris* seedlings.

**Supplementary Information:**

The online version contains supplementary material available at 10.1007/s00572-023-01135-3.

## Introduction

The genus *Tuber* (truffles sensu stricto) is the major genus of ectomycorrhizal fungi within the Tuberaceae (order Pezizales) producing hypogeous ascocarps (Paolocci et al. 2004; Læssøe and Hansen 2007). Following phylogenetic species criteria, the worldwide number of *Tuber* species is reported to be more than 200 (Bonito et al. 2010, Wei et al. 2021), of which at least 30 species occur in Europe (Stobbe et al. 2013; Molinier et al. 2016). All species of this genus are ectomycorrhizal (Agerer 1987–2012). Due to their below-ground life cycle, special features have evolved in the ascocarps, including an intense release of odor to attract spore vectors (Frank et al. 2006; Strojnik et al. 2020). Therefore, some truffles are of great economic value including *T. borchii* Vittad. (bianchetto, whitish truffle) that shares a garlic-like odor, excellent gastronomic properties, and unique aromas with *Tuber magnatum* Picco (Italian white truffle) (Zambonelli et al. 2021), as well as *Tuber melanosporum* Vittad. (Périgord black truffle), and *Tuber aestivum* Vittad. (Summer black truffle) (Hall et al. 2007; Belfiori et al. 2016). Although *T. borchii* is an excellent truffle, it has been undervalued in trading and culinary (Hall et al. 2007). Among truffles cultivated in orchards, *T. melanosporum* dominates with more than half of all traded truffles worldwide and with up to 80% of all French truffle production (Hall et al. 2003; Mello et al. 2006). While *T. melanosporum* and *T. aestivum* plantations were established worldwide wherever suitable conditions exist (Bajaj et al. 2021), *T. borchii* can be cultivated in areas where other economically important truffle species grow with less or little success (Zambonelli et al. 2002). Up to our knowledge, it is being successfully cultivated at least in New Zealand, Australia, and the USA (Zambonelli et al. 2015) due to its low host specificity and its growth in a wide range of soils (Iotti et al. 2010). In Germany, there are no known *T. borchii* plantations, and only a few were established in Europe (Belfiori et al. 2016) most likely due to commercial and cultivation dominance of *T. melanosporum* and *T. aestivum*.

*Tuber borchii* is native throughout Europe, from the Mediterranean south to as far north as central Finland, and from Ireland to Hungary in Europe (GBIF 2021). Recent records indicate presence of this species also in Iran, Middle East (Puliga et al. 2021). The species was introduced through cultivation in several countries overseas (Benucci et al. 2012; Zambonelli et al. 2015). *T. borchii* is fruiting in soils with variable carbonate content and broad pH ranging from slightly acidic (5.2) 6.5 to as basic as 7.8 (Gardin 2005; Mello et al. 2006; Hall et al. 2007). Due to its broad ecological amplitude, it can be cultivated on marginal lands (Benucci et al. 2012) with a range of plant ectomycorrhizal partners such as oaks, pines, hazel, and chestnut (Hall et al. 2007; Iotti et al. 2010). It was also successfully inoculated on *Tilia platyphyllos* (Giomaro et al. 2000) and *Carya illinoinensis* (Benucci et al. 2012), the latter one indicating possibility to grow nuts and truffles simultaneously in organic farming (Benucci et al. 2012; Freiberg et al. 2021). However, *T. borchii* prefers gymnosperm hosts (Bonito et al. 2010). Among gymnosperms, Mediterranean *Pinus* species such as *P. pinaster* and *P. pinea* are known hosts of *T. borchii* (Iotti et al. 2010) as well as synthesis of the mycorrhiza of *T. borchii* with *P. radiata* was described in pure culture (Duñabeitia et al. 1996). Prior to these studies, several successful mycorrhizations of *T. borchii* (as *Tuber albidum* Pico.) with *Pinus strobus* were realized in Italy in 1960ies and 1970es (Palenzona and Fontana 1970).

*Pinus sylvestris* is a robust tree species. Naturally, it grows on nutrient-poor, sandy soil, rocky outcrops, peat bogs, or close to the forest limit (Mátyás et al. 2004). *P. sylvestris* was also mentioned to form ectomycorrhiza with *T. borchii* (Hall et al. 2007), and the combination of these two species, both with broad ecological amplitude, makes an ideal combination for the afforestation of marginal or devastated yet not contaminated lands such as post-excavation brown coal area in Lusatia (southern Brandenburg, Germany). This approach can successfully improve socio-economic wellbeing of the people living in this area. In Germany, both species are native, *P. sylvestris* widely present (Mátyás et al. 2004), while for *T. borchii*, a personal communication with truffle collectors indicated its scattered distributed in areas Leinebergland (Lower Saxsony), Kallmuth (North Rhine Westfalia), Kaiserslautern (Rhineland Palatinate), Papproth (Brandenburg), and Gramschatzer Wald (Bavaria).

Ectomycorrhiza of *T. borchii* was morphologically and anatomically described to a different extent from several plant partners such as *Quercus* spp. (Zambonelli et al. 1993), *Pinus pinea* (Zambonelli 1995), *Pinus radiata* (Duñabeitia et al. 1996), *Pinus strobus* (Palenzona and Fontana 1970), *Corylus avellana* (Rauscher et al. 1996), *Tilia platyphyllos* (Giomaro et al. 2000), and *Carya illinoinensis* (Benucci et al. 2012). As far as we know, no comprehensive description of *T. borchii* ectomycorrhiza was published with *P. sylvestris*. Common features of all *T. borchii* ectomycorrhizas from different plant hosts are a needle-shaped long cystidia, and a puzzle-like hyphal mantle (Rauscher et al. 1996; Benucci et al. 2012). The shape of the mantle cells can vary depending on the host plant and fungal strain, ranging from polygonal to epidermoid shapes, making this ectomycorrhiza difficult to distinguish anatomically from other small white truffles or from mixed ectomycorrhiza samples (Giomaro et al. 2000). Specific primers have been developed to identify morphologically similar truffle species (Mello et al. 2006) but sequencing of the complete ITS region in nuclear ribosomal DNA is currently most reliable molecular tool to identify *T. borchii*, and to separate it from other morphologically similar small white truffles and their ectomycorrhizas (Benucci et al. 2012).

The aim of our paper was to test mycorrhizal colonization of *T. borchii* on *P. sylvestris* at different lime contents in an inoculation substrate, and to comprehensively characterize its ectomycorrhiza. In addition, we confirmed the identity of ectomycorrhiza and sporocarps, used for inoculation, by sequencing of the ITS region of in the rDNA. As far as we know, no experimental study is available investigating the effect of different lime contents on mycorrhizal parameters and growth of *P. sylvestris* seedlings in greenhouse conditions. We expected to gain information on the ecological amplitude of *T. borchii* ectomycorrhiza and the influence of pH on the mycorrhizal colonization rate.

## Material and methods

### Growth parameters and mycorrhizal colonization rate

Seedlings of *P. sylvestris* inoculated with *T. borchii* were prepared and cultivated at the German truffle nursery Trüffelbaumschule in Freiburg, Baden-Württemberg (47.997 N, 7.843 E) (www.trueffelbaumschule.de, Silke Friedrich). A spore suspension was prepared from 20 T*. borchii* ascocarps collected in region Abruzzo (Italy) and obtained from a commercial truffle dealer. Truffles were thoroughly washed, cleaned of damaged or rotten parts, frozen till preparation of spore suspension, and grinded in water prior to mycorrhization. An adequate volume of spore suspension containing 1.3 g (fresh weight) of truffles was used for mycorrhization of individual seedling. Seedlings were grown in 500 mL pots filled with a standard substrate that is regarded as trade secret of the nursery. The standard substrate was supplemented with 0 g, 5 g, 10 g, or 15 g of limestone per pot (T0, T5, T10, or T15, respectively) in form of a mixture of various CaCO_3_ particle sizes (Table 1): limestone gravel (2–8 mm), finely grounded (1–2 mm) limestone sand, and limestone dust (MilliporeSigma Cat. No. 310034). Limestone supplement was mixed in substrate well prior to beginning of the experiment to ensure adequate and uniform distribution of lime particles in pots and stabilization of pH. Germinated seeds and a spore suspension of *Tuber borchii* were added to the pots. Seedlings were kept at the truffle nursery for 9 months at a temperature of 15 °C in autumn and spring and at 25 °C in summer.

**Table 1:** Limestone forms and weights variants added to the standard inoculation substrate. pH was measured in a water suspension for each pot prior to mycorrhization and planting of seedlings

Treatment	Total limestone (g)	Limestone dust (CaCO_3)_ (g)	Limestone gravel (g)	Finely grounded limestone (g)	Substrate pH
T0	0.0	0.0	0.0	0.0	5.76 ± 0.016^a^
T5	5.0	2.0	1.5	1.5	6.32 ± 0.010^a^
T10	10.0	4.0	3.0	3.0	6.83 ± 0.019^a^
T15	15.0	6.0	4.5	4.5	7.53 ± 0.016^a^

*N* = 5 pots per treatment

At the end of experiment, pots with inoculated seedlings were transferred to the Centre for Agricultural Landscape Research in Müncheberg, Märkisch-Oderland (Germany) for immediate disassembly and measurements. For all seedlings, shoot height (from a root collar to a tip bud) was measured; shoot was removed from the roots and dried to a constant weight (air dryer, 60 °C; WTB Binder B28, Binder GmbH, Tuttlingen, Germany). Roots were gently removed from the substrate and washed under the tap water to remove the adhering substrate. Free remaining substrate from each pot was individually measured for pH (in water; pH meter HI 2223 Calibration Check pH/ORP Meter, Hanna Instruments Ltd., Leighton Buzzard, UK). All ectomycorrhiza root tips were morphologically screeded and identified following Agerer (1987–2012). All root tips and ectomycorrhiza of *T. borchii* (other mycorrhizae were not observed) were counted under a binocular microscope to calculate the mycorrhizal colonization rate. Tips of *T. borchii* with fully developed morphological characteristics were harvested for morphology and anatomy of ectomycorrhizae and for the molecular analyses. Afterwards, roots were dried using same procedure as above. Dried shoots and roots were weighted and root to shoot ratio calculated.

### Morphology and anatomy of ectomycorrhizae

Morphological and anatomical descriptions of *T. borchii* ectomycorrhizae were performed according to Rauscher et al. (1996). Descriptions are based on 20 mature ectomycorrhiza, kept fixed as described below. Anatomical preparations were investigated and measured in water. Photographs were taken with Zeiss, AxioImager Z2 microscope (Carl Zeiss Microscopy GmbH, Jena, Germany) using differential interference contrast technique.

For preparation of semi-thin sections, mycorrhizal tips were fixed in 2% glutaraldehyde in 0.1 M sodium cacodylate buffer (pH 7. 2) and kept refrigerated at +4 °C until further processing. Following six transfers in 0.1 M sodium cacodylate buffer, samples were postfixed in 1% osmium tetroxide and kept in the same buffer for 2 h in the dark at room temperature. After five washes in distilled water, samples were dehydrated in acetone line, namely, in 30%, 50%, and 70% each for 15 min, followed by 80%, 90%, and 99.9% each for 30 min, and finally, in 100% acetone, three times for 1 h. After dehydration, the samples were embedded in Spurr’s resin (Spurr 1969). Twenty mycorrhizae were cut with a diamond knife on an Ultracut Reichert ultramicrotome (W. Reichert-LABTEC, Wolfratshausen, Germany). The Sects. (0.6–0.7 µm) were stained with crystal violet for light microscopy (Zeiss Axioskop 50, Oberkochen, Germany).

### DNA extraction, amplification, and sequencing

DNA was extracted from dried truffles collected nearby Papproth (Brandenburg, Germany), from truffles used for inoculation, and from ectomycorrhizal root tips using a DNeasy Plant Mini Kit (Qiagen, Hilden, Germany). The complete nrDNA internal transcribed spacer (ITS) sequence was amplified with the primer pair ITS1/ITS4 (White et al. 1990). PCR conditions were set according to the protocol of Melanda et al. (2020). Amplification reactions were performed in a PE 9700 DNA thermocycler (Applied Biosystems, Foster City, CA, USA), with an annealing temperature of 55 °C. Before sequencing, amplified products were separated in 1.5 × agarose gel, cut from the gel, and cleaned using the Wizard SV Genomic DNA Purification System Kit (Promega Corporation, Madison, WI, USA). DNA was sequenced at Macrogen (Macrogen Europe B.V., Amsterdam, The Netherlands) with the same primers as used in PCR. Sequencher 5.4.6 (Gene Codes Corporations, Ann Arbor, MI, USA) was used to assemble consensus sequences from the two DNA strands of each isolate. The sequences obtained from the isolates were identified to the highest similarity using an online BLASTN algorithm and comparing them with those available in the GenBank database (Altschul et al. 1997).

### Statistics

Data were evaluated in Statistica (data analysis software system), version 13, TIBCO Software Inc. (2018) http://tibco.com. Growth parameters were subjected to a one-way ANOVA with a substrate treatment as a categorical factor. Equality of variances was tested by Levene test at *P* < 0.05. Where ANOVA showed statistically significant effect of substrate treatment, significant differences among groups were identified by post hoc Tukey’s HSD test. Where equality of variances was not met and could not be achieved by transformation of data, non-parametric Kruskal–Wallis test was performed. Data were considered as significantly different at *P* < 0.05.

## Results

### Pine growth parameters and mycorrhizal colonization rate

Five seedlings were measured for each substrate variant. An increased amount of lime in inoculation substrate resulted in a statistically significant increase of shoot height (*F* = 3.46, *P* = 0.0416), while other measured parameters (i.e., shoot dry weight, root dry weight, root to shoot ratio, number of root tips) were not significantly affected (Table 2). Shoots were significantly higher (*P* = 0.0304) in the T15 substrate (Table 2). Although not statistically significant, mean shoot and root dry weights were correspondingly the lowest in the T0 and the highest in the T15 substrate. pH values at the end of the experiment varied from 6.94 to 7.49 in limed pots (Table 2). All ectomycorrhizal fine roots were morphologically identified as *T. borchii*, and no other types of ectomycorrhiza were observed on tested seedlings. Individual seedlings showed highly variable mycorrhizal colonization rates ranging from 8.0 to 84.9% (both observed in treatment T5), while mean values were between 36.5 ± 2.75% in T10 and 48.1 ± 7.40% in T15. Evidently, the most variable colonization rates were encountered in T5 treatment. However, a typical and well-developed ectomycorrhizas with cystidia were only present in the treatments T0 and T5. In the T10 and T15 ectomycorrhizal root tips with few or no cystidia were formed.

**Table 2:** Growth parameters of *Pinus sylvestris* and mycorrhizal colonization with *Tuber borchii* in standard mycorrhization substrate amended with different lime quantities (T0–T15; see Table 1 for liming variants), after cultivation for one and a half years in a nursery. Results for 5 plants per treatment are given with standard error

Substrate treatment	Shoot height (cm)	Shoot dry weight (g)	Root dry weight (g)	Root to shoot ratio	Total number of root tips	Mycorrhizal colonization with *Tuber borchii* (%)	Substrate pH upon finalization
T0	12.4 ± 0.9^a^	1.20 ± 0.19^a^	0.35 ± 0.07^a^	0.28 ± 0.02^a^	880 ± 101^a^	41.2 ± 9.28^a^	6.94 ± 0.05
T5	15.7 ± 1.3^a^	1.20 ± 0.24^a^	0.30 ± 0.08^a^	0.23 ± 0.03^a^	935 ± 42^a^	41.3 ± 14.6^a^	7.23 ± 0.03
T10	15.8 ± 1.1^a^	1.39 ± 0.13^a^	0.36 ± 0.03^a^	0.26 ± 0.03^a^	1020 ± 94^a^	36.5 ± 2.75^a^	7.16 ± 0.03
T15	17.3 ± 1.0^b^	1.70 ± 0.21^a^	0.55 ± 0.11^a^	0.32 ± 0.03^a^	1230 ± 212^a^	48.0 ± 7.40^a^	7.49 ± 0.02
*F* (H)	**3.4557**	1.4886	2.2156	2.0437	2.7714	1.3086	3.0984
*P*	**0.0416**	0.2555	0.1259	0.1483	0.4282	0.7271	< 0.001

One-way ANOVA or Kruskal-Wallis tests were performed with a statistically significant difference at *P* < 0.05 and marked with different letters

Values in bold indicate significant differences among substrate treatments, at *P* < 0.05

### Morphology and anatomy of ectomycorrhizae

**Fig. 1:**
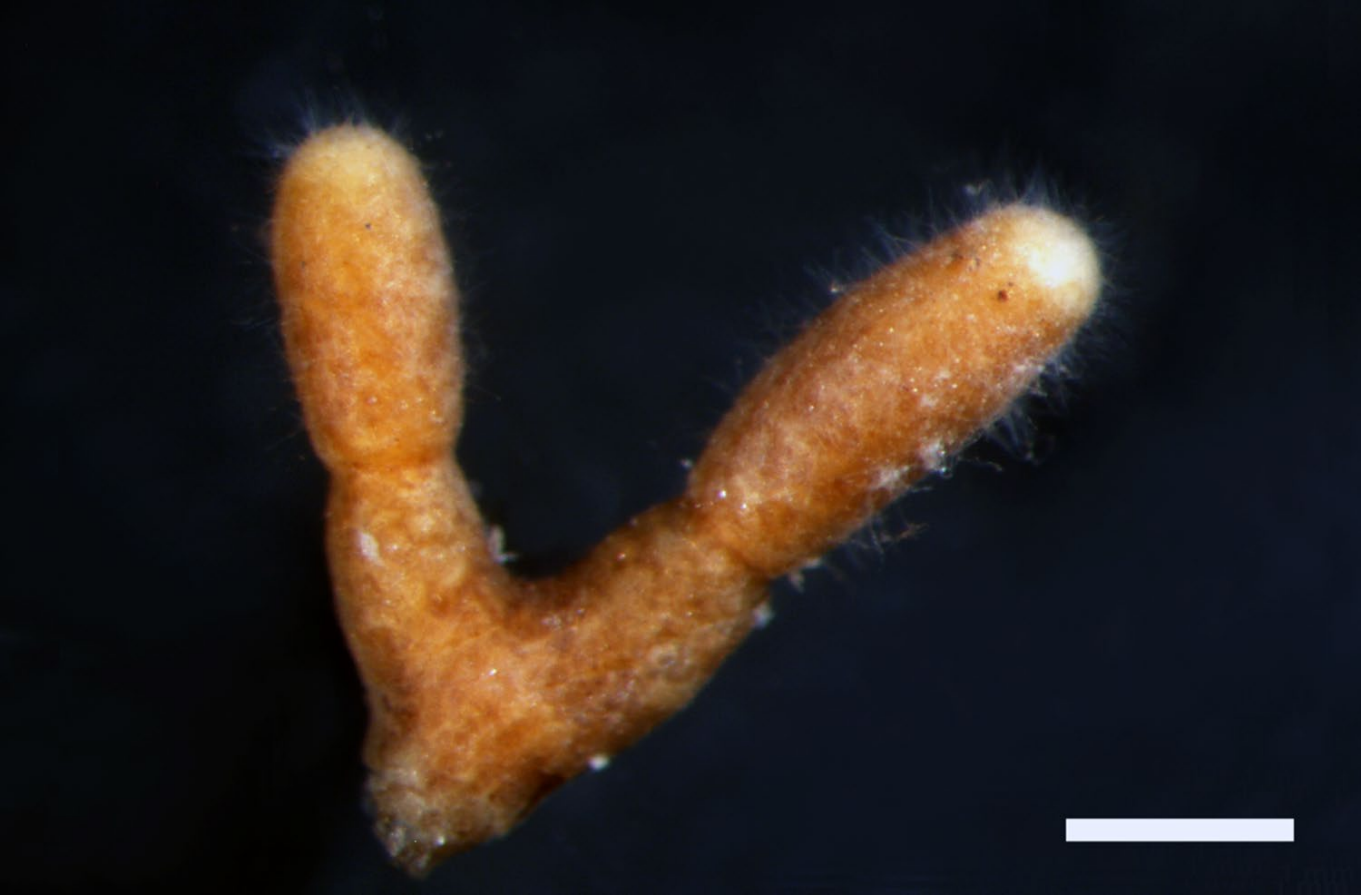
Morphology of the *Tuber borchii-Pinus sylvestris* ectomycorrhiza. Cystidia projecting from the mantle are visible. Bar = 0.5 mm

**Fig. 2:**
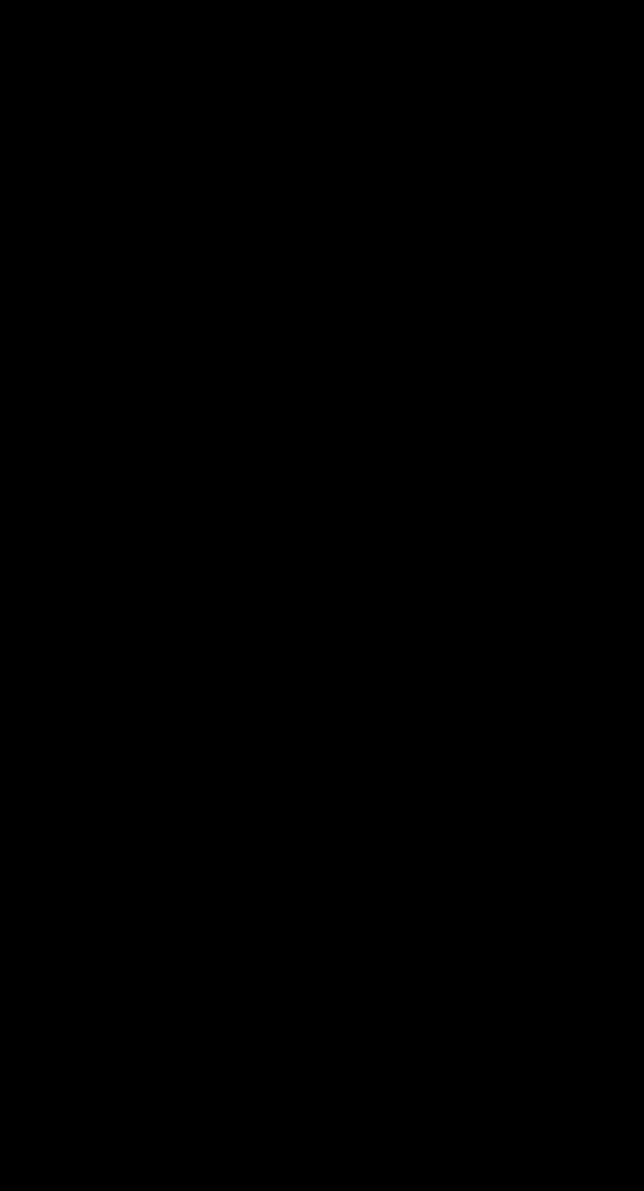
**a** Semi-thin longitudinal section of the dichotomously branched *Tuber borchii-Pinus sylvestris* ectomycorrhiza. Hyphal mantle (hm) surrounds the tip and Hartig net (Hn) grows in the basal parts of the mycorrhiza. Meristem (M) is active. CC, cortical cells; S, stele. Bar = 100 µm. **b** Magnification of the longitudinal section of the *Tuber borchii-Pinus sylvestris* ectomycorrhiza. N, nucleus; Hn, Hartig net; hm, hyphal mantle; C, cystidium. Bar = 40 µm

Ectomycorrhizal systems were dichotomously ramified or unramified. Mycorrhizae were reddish to dark brown (7.5YR 5/8 to 5YR 6/12; Munsell 1954), with growing tip of a lighter color (7.5YR 9/8 to 7.5YR 9/6; Munsell 1954). The whole ectomycorrhiza root tips from lower lime concentrations were covered by cystidia which were denser towards the root tip (Fig. 1), while scarce or no cystidia were observed in ectomycorrhiza from > 10 g lime/500 ml mycorrhization substrate (data not shown). Ectomycorrhizal hyphal mantle was composed of 3–7 (9) cell layers (Fig. 2a, b). Outer cells of the root cortex look dead and are filled with tannins, a common characteristic for pine ectomycorrhizas. In root tips where the very tip was still growing, the Hartig net was only developed in the basal parts of the ectomycorrhiza (Fig. 2a). The presence of nuclei in cortex cells proved that cells were active (Fig. 2b). Emanating hyphae, rhizomorphs, sclerotia, laticifers or excreting latex, mantle dots, or carbonizing of ectomycorrhizae were not observed.

### Anatomical characters of mantle in a plan view

**Fig. 3:**
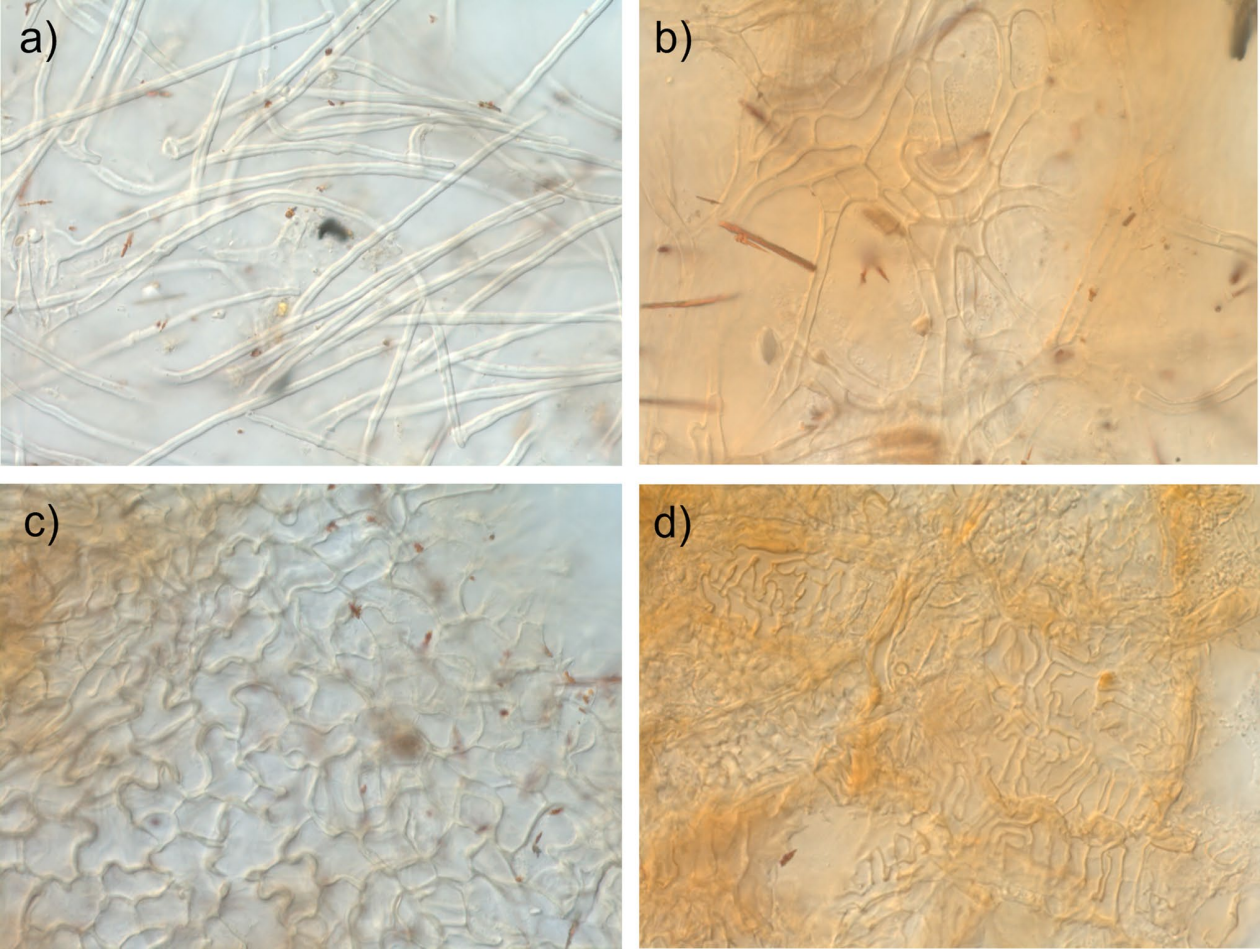
Anatomy of *Tuber borchii–Pinus sylvestris* ectomycorrhiza. **a** Cystidia. **b** Hyphal net on the outer mantle. **c** Outer mantle layer. **d** Hartig net

Mantle complete, all mantle layers are pale yellowish-brown with membranaceous position of pigment within the cell wall, cell content absent. All mantle layers are pseudoparenchymatous (Fig. 3). Cells of outer mantle layer are epidermoid to irregularly angular, bearing a hyphal net. Hyphae of hyphal net often form bundles. Cells of hyphal net 5–34 μm × 2–14 μm, hyphal cell walls distinct, 0.2–1.6 μm. Clamps are absent. Cells of outer mantle layer not specifically arranged. Cells of outer mantle layer elongated to almost roundish (3–31 μm × 2–21 μm). Cell walls 0.2–4.6 μm, smooth. Middle mantle layer pseudoparenchymatous, cells arranged without pattern, the same shape as outer mantle (3–24 μm × 2–13 μm), cell walls 0.2–2.2 μm, smooth. Inner mantle layer pseudoparenchymatous, cells arranged without pattern, same shape as outer mantle (3–39 μm × 2–13 μm), cell walls 0.2–1.5 μm, smooth. Cells of the outer mantle organized in a similar manner as other parts (1.5–20.0 μm × 1–13 μm), cell walls 0.3–2.3 μm, smooth. Hartig net with lobes 1.05–4.77 (mean 2.16 ± 0.04 μm).

### Anatomy of the emanating elements

**Fig. 4:**
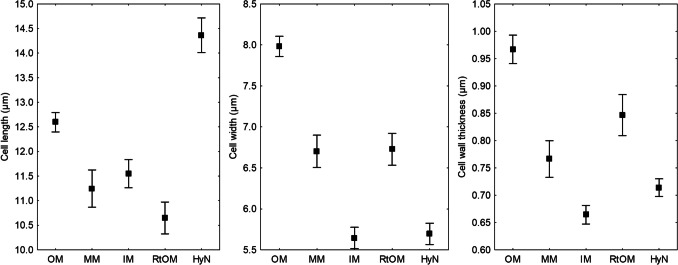
Length, width, and cell wall thickness of hyphal cells in different tissues (OM: outer mantle, MM: middle mantle, IM: inner mantle, RtOM: root tip outer mantle, HyN: hyphal net) of *Tuber borchii–Pinus sylvestris* ectomycorrhiza. Mean values ± std.err. are shown

Emanating hyphae were not observed. Cystidia originating from the hyphal net covering the outer mantle or the outer mantle itself, awl-shaped (type A), non-ramified, with 0, 1, or 2 septa at the basal part of cystidium, septa simple, foot cell absent. Length of cystidia ranging from 33 to 168 μm (mean ± std.err.: 87.4 ± 2.77 μm) (Fig. 4). Proximal diameter 2.7–8.2 μm (4.84 ± 0.07 μm), medial diameter 1.5–3.7 μm (2.51 ± 0.04 μm), distal/apical diameter 0.6–3.5 (4.6) μm (1.74 ± 0.04 μm). Cell walls 0.4–2.5 μm (0.90 ± 0.02 μm) at the base, 0.4–1.2 μm (0.82 ± 0.02 μm) in the middle, and 0.2–1.2 μm (0.42 ± 0.01 μm) at the apex. Cell walls of cystidia often covered by warts (Supplementary Fig. S1), particularly at the apex.

### Molecular analyses of fruitbodies and ectomycorrhizae

Three mature truffles collected nearby Papproth (Brandenburg, Germany), all collected within the radius of < 20 m, were sequenced, and resulted in three identical sequences (OM002609-11). Obtained sequences were 100% identical with *T. borchii* from oak dominated lowland forest in Slovenia (FM205502) and with a range of sequences published in phylogenetic or diversity studies including *T. borchii* ascocarps (Bonuso et al. 2010; Marjanović et al. 2010). Sequencing of three randomly selected ectomycorrhiza from inoculated *P. sylvestris* seedling confirmed the identity with *T. borchii* (OM0026012-14).

## Discussion

Truffle inoculation substrates are in general a producer’s secret, and only rarely information on exact composition or substrate optimisation is given (Weden et al. 2004, Freiberg et al 2021). Similarly, optimal conditions for plantation soil adaptation are broad and not necessarily adapted to a specific truffle and tree species. *P. sylvestris* inoculated with *T. borchii* was rarely used in a large-scale plantation and truffle cultivation activities. Garcia-Montero et al. (2007) even concluded that combination of *Pinus nigra* and *P. sylvestris* mycorrhized with *T. melanosporum* is unfavorable, yet some fine-tuning of conditions can yield success, as is a case in combination of joined pecan and truffle cultivation (Freiberg et al. 2021). As different truffle species have different ecological requirements (Hall et al. 2003), any information regarding adaptation of substrate composition, nutrients content, or optimal plantation soil conditions is a relevant for science and in a plantation content. *T. borchii* inoculation of *P. sylvestris* seedlings was successful regardless to the lime content used in the mycorrhization substrate and cultivation in greenhouse conditions. Mycorrhization levels did not differ significantly among different amounts of added lime while highest mycorrhization percentage average was observed for highest lime content indicating species’ high ecological plasticity in terms of lime requirements. All limestone weight variants resulted in mycorrhization level and quality, sufficient for a positive commercialization certification of seedlings (Donnini et al. 2014). pH values at the end of the experiment increased but remained in the truffle physiology range across treatments amplitude (Gardin 2005; Hall et al. 2007). Observed pH plasticity indicates that optimization of substrate with lime in case of *T. borchii*, unlike for some other host-mycorrhizal fungi, is not necessary (Freiberg et al. 2021). High mycorrhization levels, relative low effect of pH, and difference in lime content in inoculation substrate also indicate an easy greenhouse mycorrhization with *T. borchii* (Donnini et al. 2009) and confirms its broad ecological amplitude (Gardin 2005; Hall et al. 2007). However, observations from greenhouse remain to be confirmed in plantations through a successful truffle fructification. In addition, a modified plantation would be required as current management applied for truffle cultivation, such as tree pruning, could be not compatible with tree growth requirement for wood purpose. High species plasticity can explain its wide geographic distribution and occurrence in non-typical truffles sites such as devastated lands such as post-excavation brown coal area in Lusatian or high altitudinal frost areas with podzol soils (Grebenc et al. 2010).

Although *T. borchii* colonization did not significantly differ between lime treatments, the plant partner showed trends in improved growth in higher lime content in the mycorrhization substrate and cultivation in greenhouse conditions. Shoots of *P. sylvestris* seedlings were significantly higher in treatment with the highest lime addition compared to treatment with no lime addition. This can be due to quicker percolation, better retention of irrigation water through the substrate, and higher minerals weathering from gravel and lime dust content (van Schöll et al. 2006; Leake et al. 2008) in combination with an increased nitrate–N and extractable P following liming which all lead to improved productivity of the substrate (Mkhonza et al. 2020). Our findings support the idea that seedlings and mycorrhizae of *T. borchii* on *P. sylvestris* can thrive in various conditions that are often met in native habitats of *P. sylvestris* but may improve the tree growth only in conditions with higher limestone content.

Mycorrhizae of *T. borchii* are relatively well morpho-anatomically characterized from several hosts. However, different levels of accuracy in descriptions are to note, making comparison challenging. Emanating hyphae that were reported by Zambonelli et al. (1993, 1995), Rauscher (1996), and Duñabeitia et al. (1996) were not observed in our case, as they were not reported by Giomaro et al. (2000) and Benucci et al. (2012). However, hyphal net that was observed by Rauscher et al. (1996) and Duñabeitia et al. (1996) was evident. Hyphae of hyphal net in our case often occurred in bundles which is not evident from descriptions and graphics of Rauscher et al. (1996) and Duñabeitia et al. (1996). Mean length, proximal, and apical diameters of cystidia were in the range reported for *T. borchii* on *P. pinaster* (Zambonelli et al. 1995), but with greater span of values in our case. Similar lengths of cystidia for *T. borchii* were reported for *Corylus avellana*, *Quercus* (DEEMY-An Information System for Characterization and DEtermination of EctoMYcorrhizae, Agerer and Rambold 2004–2005), *Tilia platyphyllos* (Giomaro et al. 2000) and *Carya illinoinensis* (Benucci et al. 2012). On the other hand, for *T. borchii* on *P. radiata* minimum length of cystidia that is almost six times greater compared to our minimum values and maximum length that is 1.5-times of our maximum length was reported (Duñabeitia 1996). It is to note that inoculated *P. radiata* seedlings were grown in pure culture on Melin-Norkrans medium, whereas our report is from an inoculation substrate grown ectomycorrhizae. In our case, well-formed mycorrhiza with cystidia were only found in the T0 and T5 substrate treatments. This may be due to better irrigation of the substrates, since limestone gravel and finely grounded limestone are quantitatively higher in the T10 and T15 treatments. Thus, the flow rate and distribution of water could be better in these treatments. On the other hand, the absence of the cystidia could be an ontogenetical stage. Very young cultures of *P. sylvestris-T. borchii* ectomycorrhiza from the greenhouse showed a lack of cystidia on their mycorrhizae while older cultures bore plenty *T. borchii* mycorrhizae with well-developed cystidia. Therefore, we believe that the lack of cystidia is due to the ontogenetical stage of the mycorrhizae.

Surface of cystidia was covered by warts, so far only reported for *Corylus* (Rauscher et al. 1996). Warts were also described for *Tuber magnatum*, another among the white truffles (Mello et al. 2001). Cystidia contained 0–2 septa, and the same number of septa was also reported by Giomaro et al. (2000). However, one of the strains investigated by Giomaro et al. (2000) contained three septa.

Mean length of cells in outer mantle layer was slightly lower compared to the reports of Giomaro et al. (2000) and Benucci et al. (2012). Compared to the description of Rauscher et al. (1996) who noted mantle cell walls thickness of 1.0 µm in outer layer, and up to 0.5 µm in inner layer, our samples revealed greater span of cell wall thickness in all mantle layers, i.e., 0.2–4.6 μm in outer, 0.2–2.2 μm in middle, and 0.3–2.3 μm in inner mantle layer. As evident, even in the middle and inner mantle layer, some cell walls were relatively thick.

As reported for different strains of *T. borchii*, cell shape of outer mantle may vary from polygonal with rounded edges to epidermoid with marked lobes (Giomaro et al. 2000). Variable cell shape was also reported for *T. borchii* from *Carya illinoinensis*, from ellipsoid and isodiametric to irregular-rectangular and elongated (Benucci et al. 2012). Shapes of cells were variable also in our case, but with prevailing epidermoid type. Giomaro et al. (2000) pointed out that solely the outer mantle organization cannot be used to distinguish among ectomycorrhizae from different species in the “white truffles.”

## Conclusions

The mycorrhization of *P. sylvestris* with *T. borchii* in the mycorrhization substrate and cultivation in greenhouse conditions was successful. Lime and consequently pH did not affect mycorrhization levels, but it improved growth of the *P. sylvestris* seedlings. With our study, it was shown that *T. borchii* can form ectomycorrhizae with *P. sylvestris* in substrates with different amounts of limestone, therefore, making it a potential candidate for a wide variety of substrates. Although the colonization rates were insignificantly different among treatments, the carbonate content might be decisive in future developmental steps including sporocarp production of other truffle species as is a case in *T. melanosporum* (Garcia-Montero et al. 2012). Opposite to mycorrhization, we have shown that improved growth of *P. sylvestris* seedlings occurred only in substrate with higher limestone contents. Morpho-anatomical properties of *T. borchii* on *P. sylvestris* are similar as described for *T. borchii* on other tree species, with the exception that hyphae of hyphal net occurred in bundles.

### Supplementary Information

Below is the link to the electronic supplementary material.

Supplementary file1 (DOCX 1280 KB)

## Data Availability

The raw data is deposited in the public nucleotide database or is available at the first author.
