# Supplementary material for: Description, identification, and growth of Tuber borchii Vittad. mycorrhized Pinus sylvestris L. seedlings on different lime contents
Source: Mycorrhiza. 2024 Jan 18;34(1-2):85–94. doi: 10.1007/s00572-023-01135-3 (PMC10998771; doi:10.1007/s00572-023-01135-3)
Supplement: Supplementary file 1 — Supplementary file1 (DOCX 1280 KB) [file 572_2023_1135_MOESM1_ESM.docx]

Description, identification, and growth on different lime contents of mycorrhizal *Tuber borchii* Vitt. and *Pinus sylvestris* L.

Tanja Mrak^1^, Tine Grebenc^1^, Silke Friedrich^2^ and Babette Münzenberger^3^

^1^Department of Forest Physiology and Genetics, Slovenian Forestry Institute, Večna pot 2, 1000 Ljubljana, Slovenia

^2^Truffle nursery, Schneckleinsberg 5, 91788 Pappenheim, Germany

^3^Department of Fungal Interactions, Research Area 1 ‚Landscape Functioning‘, Leibniz Centre for Agricultural Landscape Research (ZALF), Eberswalder Strasse 84, 15374 Müncheberg, Germany

Supplementary file


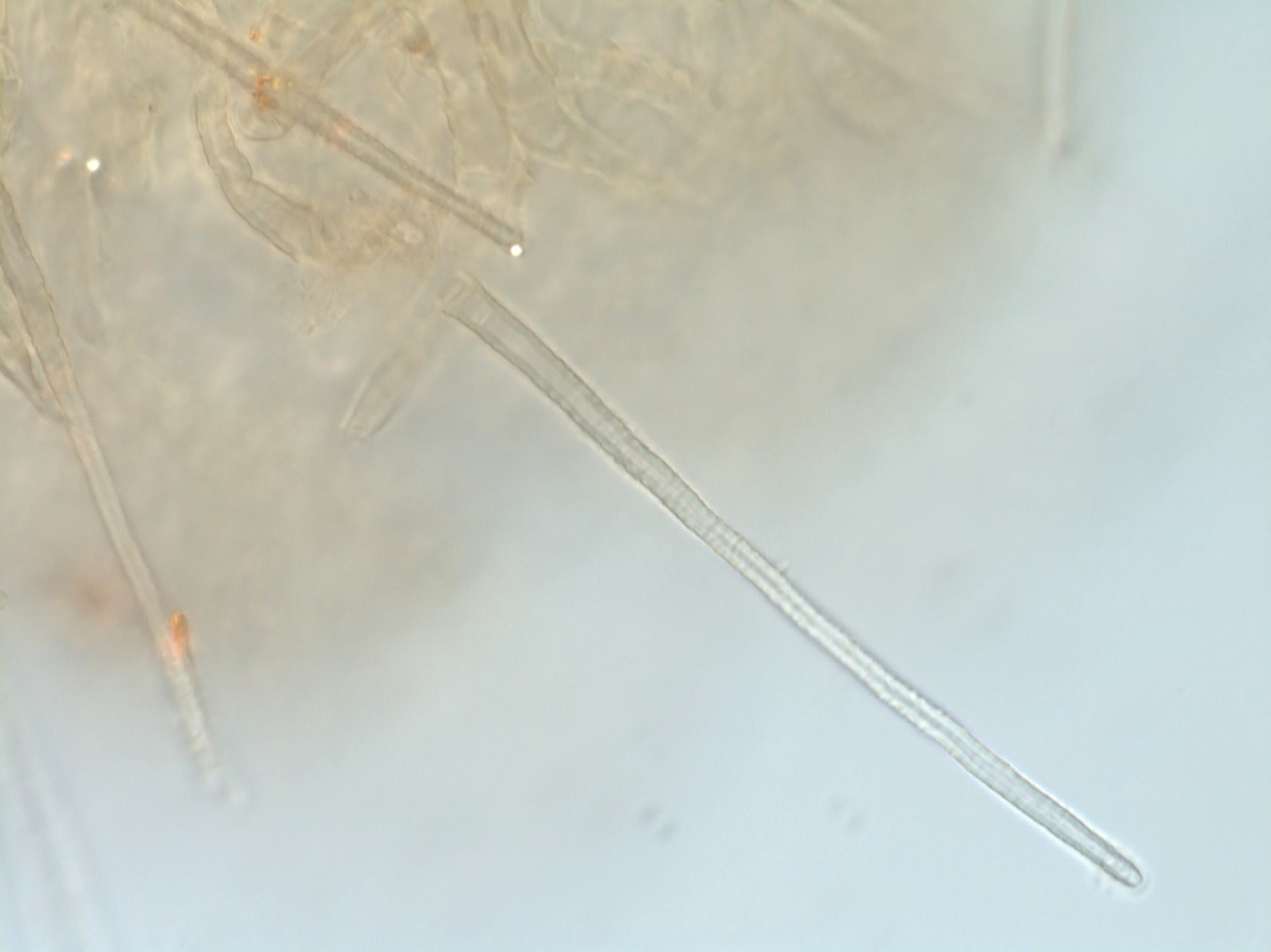


Figure S1: Warts covering cystidia of *Tuber borchii* ectomycorrhizae.
